# The neural system regulates bone homeostasis via mesenchymal stem cells: a translational approach

**DOI:** 10.7150/thno.43771

**Published:** 2020-03-26

**Authors:** Xu-Dong Wang, Si-Yi Li, Shi-Jian Zhang, Anand Gupta, Chen-Ping Zhang, Lei Wang

**Affiliations:** 1 Department of Oral and Maxillofacial-Head & Neck Oncology, Shanghai Ninth People's Hospital, College of Stomatology, Shanghai Jiao Tong University School of Medicine; National Clinical Research Center for Oral Diseases; Shanghai Key Laboratory of Stomatology & Shanghai Research Institute of Stomatology, Shanghai, China; 2 Department of Dentistry, Government Medical College & Hospital, Chandigarh, India

**Keywords:** bone marrow mesenchymal stem cell, bone homeostasis, peripheral nerves, bone regeneration, bone graft

## Abstract

Large bone reconstruction is a major clinical issue associated with several challenges, and autograft is the main method for reconstructing large defects of maxillofacial bone. However, postoperative osteoporosis of the bone graft, even with sufficient vascularization, remains a primary problem. Therefore, better understanding of the mechanisms and clinical translation of bone homeostasis is required. Neuronal innervation of the bone is an emerging research topic, especially with regards to the role of peripheral nerves in regulating bone homeostasis. Moreover, sensory and autonomic nerves regulate this process *via* different types of neurotransmitters, but the specific mechanism is still elusive. In this review article, the current understanding of the interaction between the peripheral nerve and the skeleton system is summarized, with a particular focus on bone marrow mesenchymal stem cells (BMMSCs), except for osteoblasts and osteoclasts. The novel application of nerve-based bone regeneration *via* BMMSCs may provide a new strategy in tissue engineering and clinical treatment of osteoporosis and bone disorders.

## Introduction

Large defects of the maxillofacial bone caused by tumors, trauma and congenital malformation, etc., seriously affect the appearance and function of the patient. Moreover, functional reconstruction is clinically difficult, but is highly important. Tissue engineering is a promising technology, with previous cutting-edge studies showing the importance of advanced scaffold materials with controlled release or molecularly imprinted intelligence for tissue regeneration or cancer treatment 1-6, using biomaterial alignment and optimized mechanical stimulation driven by the differentiation of mesenchymal stem cells (MSCs) 7. Autograft remains the main method for reconstructing large continuous defects of the maxillofacial bone, but severe postoperative osteoporosis can be found after non-vascularized bone grafts 8-11. Although the effect of free vascularized bone grafts is higher compared with non-vascularized bone grafts, the postoperative spontaneous osteoporosis of the bone graft is severe and hinders the success of dental implants, even after microsurgical vascularization 8, 11, 12. Currently, there is no effective preventive method and the underlying mechanism remains unknown. Hence, this has become a focus of the reparative and reconstructive surgery research field.

Based on the fact that postoperative osteoporosis, despite a sufficient blood supply, inevitably develops in autografted bones indicates that there may be systemic factors other than just blood supply that control the internal environment of the bone graft. As the blood supply of bone is accompanied by innervation, it has been hypothesized that nerves play an important role in regulating the bone homeostasis. Previous studies have revealed that sympathetic nerves and sensory nerves affect bone metabolism and bone remodeling *via* certain neurotransmitters 13-16. Furthermore, it has been demonstrated that the peripheral nervous system is involved in bone metabolism, osteogenic differentiation, bone mineralization and bone remodeling. Our previous studies have demonstrated that the sympathetic nervous system (SNS) inhibits bone remodeling by inhibiting bone marrow mesenchymal stem cells (BMMSCs), as well as indicating that, Nerve Growth Factor (NGF) and Substance P (SP) can promote bone formation *via* sensory nerves 17-20. Therefore, based on the results of animal experiments and necropsy, our research group has investigated in the development of a vascularized iliac bone grafting method with simultaneous innervation using neurorrhaphy between the nerves innervating the iliac bone and recipient site. Our clinical retrospective study suggested that this novel method significantly decreases postoperative osteoporosis and improves the success of dental implants 21. Moreover, a previous tissue engineering study has found that in addition to vascularization, innervation may also play an important role in promoting tissue engineered bone formation 22.

Remodeling of innervation effectively prevents osteoporosis of the bone graft, suggesting that the mechanism *via* which innervation regulates bone homeostasis may involve BMMSCs. Both sympathetic and sensory nerves play a crucial role in regulating bone remodeling *via* specific neurotransmitters. In particular, sensory neurotransmitters, such as NGF, Calcitonin Gene-related Peptide (CGRP), SP, and Semaphorin 3A (Sema3A) positively regulate bone formation *via* BMMSCs. The present review examines how peripheral nerve regulates bone homeostasis *via* MSCs, which may provide a basis for translational research of systemically regulating bone remodeling, regeneration and prevention of osteoporosis.

## Relationship between bone homeostasis and 'aging' BMMSCs in osteoporosis

Osteoporosis is a very common skeletal degenerative disease in the elderly and menopausal women. Moreover, it is characterized by decreased bone mineral density (BMD) and destruction of bone microarchitecture, which give rise to an increased risk of fragility fracture of by more than 40% 23, 24. Therefore, there is thus an urgent need for novel therapies to not only reduce the risk of fracture, but also to prevent the active bone loss in early disease phases. Furthermore, it is essential to identify the mechanism of osteoporosis, which may be caused by an imbalance in bone formation and resorption 23.

Osteoporosis is related to 'bone homeostasis', which mainly refers to the relatively stable state of the intraosseous environment under the precise regulation of the system network, which should be the bone remodeling balance between osteogenic activity of osteoblasts and bone resorption activity of osteoclasts. In addition, osteoporosis occurs when the activity of osteoblasts decreases and that of osteoclasts increases, due to the disturbed balance in bone homeostasis.

Bone homeostasis is closely related to the 'aging' of BMMSCs. Friedenstein et al. 25 identified a group of cells with osteogenic potential that were derived from bone marrow, and had a morphotype similar to fibroblasts, displaying rapid adherence to tissue culture vessels. These cells are referred to as 'Mesenchymal stem cells (MSCs)' 26. MSCs are defined as self-renewable, multipotent progenitor cells with the capacity to differentiate into several distinct mesenchymal lineages, and are uniformly positive for CD29, CD44, CD71, CD90, CD106, CD120a, CD124, amongst other surface proteins 27. BMMSCs, the mesenchymal progenitors for both osteoblasts and adipocytes, have two features: self-renewal and a multipotent ability 28, 29. Aging is characterized by common environmental changes, such as hormonal, immunological and metabolic disorders. Moreover, 'aged' BMMSCs refer to dysfunctional cells, with differentiations shifting toward adipogenesis rather than osteogenesis 30. Furthermore, in an osteoporosis mouse model BMMSCs are shown to display an 'aging' phenomenon, resulting in decreased osteogenic differentiation ability, enhanced adipogenic capacity, decreased self-renewal ability and decreased ability of induced-apoptosis in osteoclasts and T cells, ultimately leading to unbalanced bone homeostasis 31-33. Therefore, 'aging' BMMSCs may be an important factor involved in unbalanced bone graft homeostasis, and thus further examination of the promotion of osteogenesis differentiation should be considered. Moreover, the osteogenic activity of BMMSCs should be present in both autograft and tissue-engineered bones to avoid postoperative absorption.

BMMSCs, as key cells for bone regeneration and maintenance, have become a hot topic of research in recent years. Furthermore, there are ongoing clinical trials for treating non-union of the bone, osteonecrosis of the femoral head and other bone diseases by directly injecting autologous MSCs or implanting with carriers 34. In addition, studies using stem-cell-based regeneration of bone with bone tissue engineering grafts and growth factors have been successful 34. A previous study used this method with bone marrow aspirates and customized titanium cage culturing in *latissimus dorsi* muscle to regenerate mandibular bone, and the results provide a good example of ectopic bone formation 35. Since additional surgery increases the risk of complications in the donor site, dental implants can be inserted into the new bone in *in-situ* bone formation with autologous adipose stem cells and β-tricalcium phosphate granules 36. It has been demonstrated that BMMSCs promote cartilage regeneration to treat osteoarthritis *in vitro* 37. Interestingly, MSC-based immunotherapy using systemic infusion has beneficial effects in patients with graft-versus-host disease 38. Moreover, recipient glycemic micro-environments result in enhanced effects of BMMSCs therapy following BMMSCs infusion in an experimental osteopenia model 39. In addition, systemic transplantation of stem cells from human exfoliated deciduous teeth has been used to treat systemic lupus erythematosus in mice 40. MSCs therapies in different diseases, including heart failure and enterocutaneous perianal fistular disease have made progress, but standardizing MSC preparation, fitness and functionality remains difficult 41.

## Microenvironmental regulation of BMMSCs

Stem cells are controlled by a microenvironment called the 'stem cell niche', rather than existing independently 42. Furthermore, there has been increasing attention on how the systematic microenvironment regulates the stem cells. It has been shown that osteoblasts differentiate from BMMSCs, while osteoclasts differentiate from hematopoietic stem cells (HSCs), and both BMMSCs and HSCs coexist in the microenvironment of interacting stem cells 42. Moreover, the interaction of BMMSCs with cells differentiated from HSCs, including T cells, natural killer cells and osteoclasts, influences bone remodeling 43. The role of T cells in bone remodeling was first identified in 1983, where it was found that bone resorption occurred in T-cell-deficient nude mice 44. Furthermore, in an osteoporosis model, T cells are demonstrated to induce apoptosis of BMMSCs and osteoblasts *via* the CD40/CD40L pathway 43. It has also been shown that BMMSCs express Fas ligand (FASL) and can recruit activated T cells to promote apoptosis 42, 45, 46. Moreover, cytotoxic T‑lymphocyte protein 4 (CTLA-4), which is secreted by regulatory T cells, can bind to CD80/CD86 on osteoclast precursors to promote apoptosis and reduce bone resorption 47. Receptor activator of NF-κΒ ligand (RANKL) was first discovered to regulate T cell differentiation and is an important factor in osteoclast differentiation 48. It has also been demonstrated that osteoprotegerin (OPG) is an osteoclast differentiation inhibitor, and serves as a decoy receptor of RANKL. Furthermore, B cells can secrete OPG to inhibit RANK-RANKL binding, which slows osteoclast differentiation and stabilize bone mass 49.

Previous studies have shown that immune cells can secrete pro-inflammatory factors that damage BMMSCs and affect tissue regeneration 50, 51. For example, HSC-derived lymphocytes secrete interferon (IFN)-γ and tumor necrosis factor (TNF)-α to inhibit BMMSC differentiation 43, 47. Using an ovariectomy-induced osteoporotic mice *in vitro* model, our previous study reported that inflammatory microenvironment can cause loss of differentiation potential of normal BMMSC and apoptosis but can also lead to a reduced ability of BMMSC to induce apoptosis of osteoclasts 32, 33, 46. Moreover, TNF-α and IFN-γ negatively regulate the osteogenic capacity of BMMSC *via* the NFκB and Wnt pathways, and the alteration of these pathways affects runt-related transcription factor 2 (Runx2) and peroxisome proliferator-activated receptor γ (PPARγ), which are two key transcription factors regulating osteogenesis and adipogenesis, and thus causes stem cell aging 30, 32. Bone marrow stromal cells (BMSCs), differentiated from BMMSCs, can either secrete RANKL, which binds RANK on osteoclast precursor cells, or secrete macrophage - colony stimulating factor (M-CSF), which binds to its receptors expressed by osteoclast precursors, thus promoting osteoclast differentiation 47.

As nerve fibers are involved in the formation of the bone marrow stem cell niche, the regulation of stem cells by the nervous system has become an increasing focus of research 42. Furthermore, a previous study suggested a role of the sympathetic system in regulating mobilization of HSC to the stem cell niche 13. Zhao et al*.* 52 first discovered the presence of a MSC niche around a neurovascular bundle in the mouse incisor model and demonstrated that the vascular nerve bundle regulates MSC homeostasis by secreting Sonic Hedgehog protein. Previous studies using tissue engineering have shown that innervation, in addition to vascularization, may also play a crucial role in promoting tissue engineered bone formation 22, 53. Moreover, it has been revealed that adipocytes regulate various tissues by transmitting signals to local nerve fibers 54. The study of neurotransmitters, such as leptin, CGRP, SP and Sema3A, on bone homeostasis has been increasing 55, 56, with a focus on examining, how various neurotransmitters regulate 'aging' stem cells and may reduce osteogenic capacity.

## Innervation of bone

According to Hilton's rule 57, nerves innervating the muscles also innervate the attached bones. In addition, large nerve bundles accompanying blood vessels nourish bones at different locations. Therefore, the relationship between the innervation of sensory nerves and autonomic nerves and the regulation of bone homeostasis has attracted increasing attention. A large number of sensory nerve fibers, which are sensitive to mechanical stress and pain, innervate the trabecular bone and periosteum 58, 59. Furthermore, it has been shown that these sensory nerve fibers promote osteogenesis by secreting sensory neurotransmitters, such as NGF, CGRP and SP 55, 60; although detailed understanding of their function in different tissues remains elusive (Table 1). The autonomic nervous system is divided anatomically and functionally into two opposed arms, the SNS and the parasympathetic nervous system (PSNS). The SNS plays a crucial role in the connection between the central control and terminal effectors. Furthermore, norepinephrine (NE) is a major neurotransmitter of the SNS and inhibits bone formation by stimulating α- and β-receptors, while Neuropeptide Y (NPY) is regarded as an inhibitor of NE 55, 61. Moreover, the parasympathetic neurotransmitter acetylcholine (ACh) plays a role in bone protection by activating muscarinic and nicotinic cholinergic receptors 61. However, the precise distribution and density of the SNS and PSNS in bone are not fully understood. In general, preganglionic neurons are cholinergic, while postganglionic neurons are noradrenergic. In addition, sympathetic postganglionic neurons cover the majority of tissues in the body, along with major nerves that contain predominantly sensory and somatic motor nerve fibers.

## Regulation of BMMSC by different nerve fibers *via* neurotransmitters

**Figure 1:**
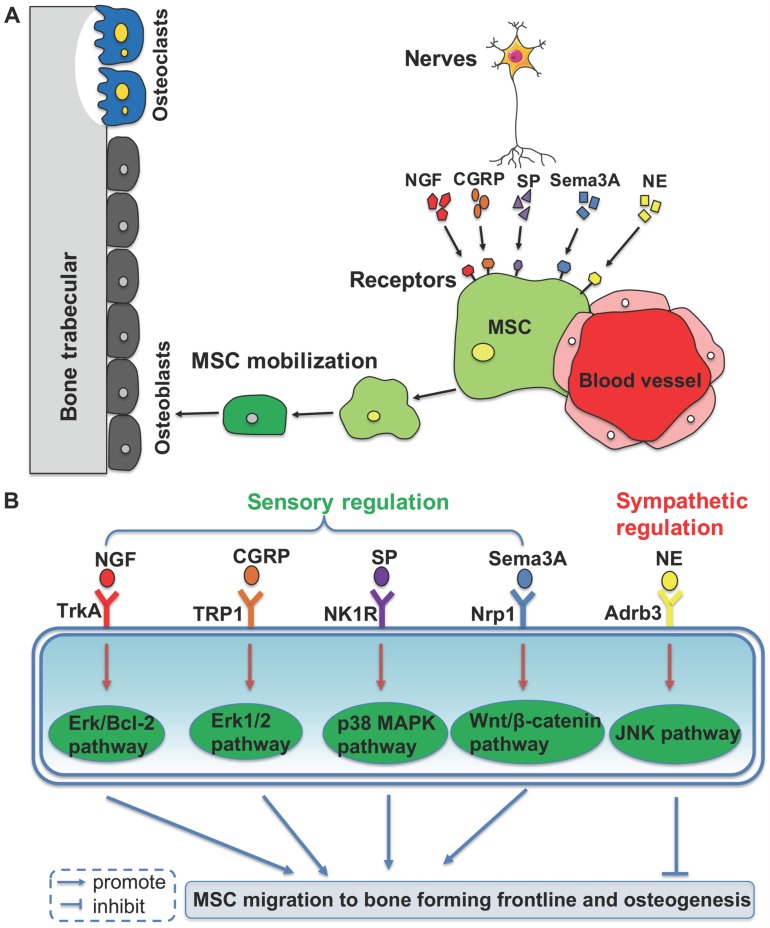
** Peripheral nerves regulate mobilization and differentiation of mesenchymal stem cells (MSCs) from their perivascular niche to frontlines of osteogenesis, *via* different molecular pathways. (A)** Neurotransmitters binding to the membrane receptors trigger the intracellular pathways to promote or inhibit MSC migration to the bone forming sites. **(B)** It shows the detailed molecular pathways.

Based on the clinical discovery that patients who are paralyzed are prone to osteoporosis, the role of the nervous system in maintaining bone mass in bone metabolism has become an important research topic 62. It has been revealed that there is a close relationship between the central nervous system and bone metabolism. Leptin from the hypothalamus is related to osteogenesis *via* stimulation from the sympathetic nerves and it can inhibit bone formation *via* the β2 receptor expressed on osteoblasts 15. A recent study has demonstrated that leptin can also act as a physiologic signal to leptin receptor expressed by BMMSCs, thus inhibiting osteogenesis and inducing adipogenesis 63. Moreover, sensory and sympathetic nerves promote the migration of BMMSCs to the osteogenesis front line by using different neurotransmitters, which maintains an active bone environment in osteogenesis (Figure 1).

### NGF

NGF is a member of the neurotrophic factor family and plays a critical role in the development of various types of nerve cells in the central and peripheral nervous systems 64. It has been shown that NGF increases osteoblast differentiation, proliferation and activity, and is also involved in osteoclastogenesis 65, 66. Our previous study reported that NGF can promote the recovery of mandibular sensory nerves in distraction osteogenesis (DO) and indirectly promote bone regeneration 19, 20. In addition, our previous study found that, in the rabbit mandibular DO model, the local application of NGF during the consolidation period can accelerate wound healing to shorten this period 20. Our previous results also suggested that using Collagen/nano-hydroxyapatite/kappacarrageenangels to inject NGF at the DO region can achieve improved bone-promoting effects 19. Moreover, NGF promotes osteoblast differentiation and inhibits its apoptosis for bone remodeling, as well as accelerates the recovery of inferior alveolar nerve in DO 67, 68. It has been revealed that NGF directly regulates wound repair during bone fracture healing by activating the NGF/TrkA signaling pathway, which causes load-induced nerve sprouting, particularly in the early stage 66. A previous study also showed that NGF binding to TrkA enhances the survival and regenerative capacity of bone marrow stromal stem cells *via* the upregulation of the Erk/ Bcl-2 pathway 69. Collectively, these findings suggest that NGF promotes bone regeneration and reconstruction.

### CGRP

CGRP is a 37 amino acid peptide synthesized by the CGRP gene on the short arm of chromosome 11 and is widely found in the central and peripheral nervous systems 14. In the maxillofacial region, CGRP is secreted from the trigeminal semilunar neuron and is transported to the bone and other effector tissues *via* the trigeminal axons, and can perform its biological effects by binding to the main receptor transient receptor potential vanilloid 1 (TRPV1). Moreover, the effect of CGRP in bone metabolism has been demonstrated in osteoporosis-phenotype CGRP-knockout mice 14. In addition, our previous study revealed that numerous CGRP-based neuropeptides promote mobilization and osteogenic differentiation of BMMSCs in jaw regeneration 70.

CGRP can act on various intraosseous cells such as osteoblasts and osteoclasts. Furthermore, it has been shown that CGRP inhibits osteoclasts *via* RANKL/OPG pathways under the activation of TRPV1, resulting in reduced bone resorption in animal models with experimental periodontitis 71. Previous studies have also revealed that CGRP receptors are expressed on osteoclasts and that CGRP can inhibit osteoclastogenesis *in vivo* and *in vitro* 72-74, especially inhibiting the differentiation of early osteoclasts 71. The p38 signaling pathway is important for regulation of BMMSC osteogenesis, and our previous study demonstrated that jaw-derived BMMSCs mobilize to the osteogenesis front line and play an important role in osteogenesis under the effect of static strain activated p38-MAPK signaling 70, 75. Therefore, these results provide experimental evidence for enhancing DO efficiency. Moreover, based on these findings it was speculated that the CGRP neuropeptide stops the 'aging' BMMSC microenvironment, but further studies are required to investigate the potential involvement of other pathways in which CGRP may participate.

### SP

SP, a neuropeptide of the tachykinin family, is a highly conserved 11-amino acid neuropeptide involved in pain perception, and has identical protein sequences in mice, rabbits and humans 76. SP mainly binds to the neurokinin 1 receptor (NK1R) on non-neuronal cells, including BMSCs, BMMSCs, osteoblasts and osteoclasts, and thus regulates both osteogenesis and osteoclastogenesis 77. Furthermore, SP can stimulate osteoblast osteogenesis *via* NK1R in advanced bone formation. It has also been demonstrated that in skull osteoblast SP induces increased mineralization and expression levels of the bone-related proteins Runx2 and osteocalcin 78. SP stimulation also promotes osteoclastogenesis of isolated bone marrow macrophages and bone resorption activity of mature osteoclasts 14, 78, 79. It has been reported that RANKL-induced cytosolic free Ca2^+^ signaling accelerates NF-κB nuclear translocation in osteoclasts, and SP activates NF-κB in bone marrow macrophages (BMMs), which directly facilitates RANKL-induced macrophage osteoclastogenesis and bone resorption activity 77, 80. Moreover, SP stimulates BMSCs to produce RANKL, but at concentrations that are too low to evoke osteoclastogenesis 77. Furthermore, a lack of SP may lead to a decrease in bone resorption rate, as well as late bone formation and mineralization rate, resulting in a net bone loss due to a greater rate of bone resorption than bone formation 79, 81.

In addition, previous studies have shown that SP can stimulate the proliferation of BMMSCs and the mineralization of differentiated BMMSCs *in vitro* 77, 81. It has been reported that SP facilitates the proliferation of BMMSC in a concentration-dependent manner: high concentrations of SP stimulate BMMSC proliferation and mineralization, while low concentrations of SP stimulate osteoblast differentiation at a later stage 77. Our previous study also demonstrated that local injection of SP in a rat mandibular DO model can accelerate bone remodeling and bone maturity, and also increases MSC migration during osteogenesis 18. Furthermore, systemic injection of SP increases the migration of CD29^+^ MSCs to the wound region, which accelerates bone remodeling and bone maturation by activating the Erk1/2 signaling pathway 18, 76. Collectively, SP stimulates the migration of BMMSCs to the osteogenesis area and promotes differentiation to increase mineralization, thus leading to bone formation. However, the appropriate concentration and signaling pathways involved in the process requires further examinations. SP can also stimulate immune cells to secrete pro-inflammatory factors, and thus acts as a mediator of adipose tissue inflammation, leading to metabolic dysfunction 54.

### Sema3A

Sema3A, an axon guidance molecule, belongs to the semaphorin family and is an important member of the vertebrate sensory neurotransmitter microenvironment. In addition, Sema3A is a membrane-associated secretory protein in the central nervous system, which is involved in guiding axonal and neuronal migration 82. It has been shown that Sema3A is also involved in organogenesis and angiogenesis 83. Fukuda et al. 84 were the first to discover the positive role of sensory nerves in bone remodeling, and reported a low bone mass phenotype in a neuron-specific Sema3A-deficient mouse model, but normal bone formation and bone mass in osteoblast-specific Sema3A-deficient mice 84. Thus, high expression levels of Sema3A in bone may be derived from neurons, and a decrease in Sema3A expression in nerves innervating the intraosseous is a major factor in the reduction of bone formation and increase of bone resorption. Therefore, it was speculated that Sema3A may regulate bone metabolism *via* peripheral nerves.

Neuropilin 1 (Nrp1), the main receptor for Sema3A, is a protein encoded by the *npr1* gene, which is located at 10p11.22 and plays a broad role in angiogenesis, axon guidance, cell survival, migration and invasion. Another receptor of Sema3A Plexin A1 (PlxnA1) forms a complex with Nrp1 to protect bone 85. Furthermore, Sema3A binds to Nrp1 to mediate signal transduction, activate osteoblast differentiation, and inhibit adipocyte differentiation, osteoclast precursor migration and osteoclast differentiation 85, 86. Nrp1 has two complement binding domains, two coagulation factor domains and one MAM domain. Moreover, Sema3A binding to the extracellular complement-binding domain causes conformational changes of PlxnA1 and activates intracellular signals to promote osteogenic differentiation 87. It has been demonstrated that expression of Sema3A in bone participates in not only innervation but also regulation of vascular invasion, which may contribute to bone formation.

The majority of the factors regulate bone homeostasis from a single aspect, while Sema3A both inhibits bone resorption and increases bone formation to protect bone 85. Competing with TREM2, Nrp1 binds to PlxnA1 to inhibit both osteoclast differentiation pathways ITAM and RhoA and promote the Wnt osteogenic differentiation pathway 85. In addition, Sema3A stimulation upregulates Nrp1 expression, causing BMMSCs differentiation into osteoblasts 85. It has also been shown that Sema3A binds to the Nrp1 receptor and specifically activates Rac1 mediated by FARP2, which leads to the accumulation of Wnt3a-activated β-catenin in the nucleus, ultimately resulting in increased osteogenic differentiation and reduced adipogenic differentiation 88. Such increased osteogenic differentiation can be contributed by promoting osteogenesis genes such as RUNX2, whereas reduced adipogenic differentiation can be caused by inhibiting lipogenic genes such as PPAR-γ 87, 89. Furthermore, the self-renewal and osteogenic differentiation potential of BMMSCs is determined by the expression level of Wnt3a 89. Previous studies have reported that the expression of the osteogenesis-associated gene RUNX2 is increased in stem cells that have high expression of Sema3A 90. Thus, Sema3A signaling is important for neuronal targeting in the peripheral nervous system. In addition, neuronal-derived Sema3A can act as an autocrine factor to promote the normal development of the nervous system 84, 91.

### NE

The negative regulation of bone remodeling by SNS has also been previously reported 55, 56, 61. NE, as a main neurotransmitter of SNS, participates in the regulation of bone homeostasis mainly *via* its β2 receptor, which stimulates osteoclast formation leading to bone resorption 92, 93. With regards to its underlying mechanism, noradrenergic nerve terminals in bone release NE to stimulate β2-AR expressed on osteoblasts and osteocytes. This activation leads to an increase in RANKL expression, reduced bone formation and increased bone resorption 56. In contrast, the expression levels of the β1 and β3 receptors on osteoblast cell lines are weak to nondetectable 56. Furthermore, application of β-blockers can reduce fracture risk 55, 94. Our previous studies reported that sympathetic nerves mainly play a negative regulatory role. In addition, the resection of sympathetic nerves down-regulates NE-β3 receptor expression and distraction stress can promote local BMMSCs, causing sympathetic nerve and endothelial stem cells to migrate to the osteogenesis front line *via* Stromal cell derived factor 1 (SDF-1), matrix metalloproteinase 2 (MMP-2) and tissue inhibitor of metalloproteinase 3 (TIMP-3). Moreover, this process results in decreased bone mass *via* the NE/abrd3/JNK pathway 17, 95.

## Clinical studies using nerve-supported bone homeostasis

**Figure 2:**
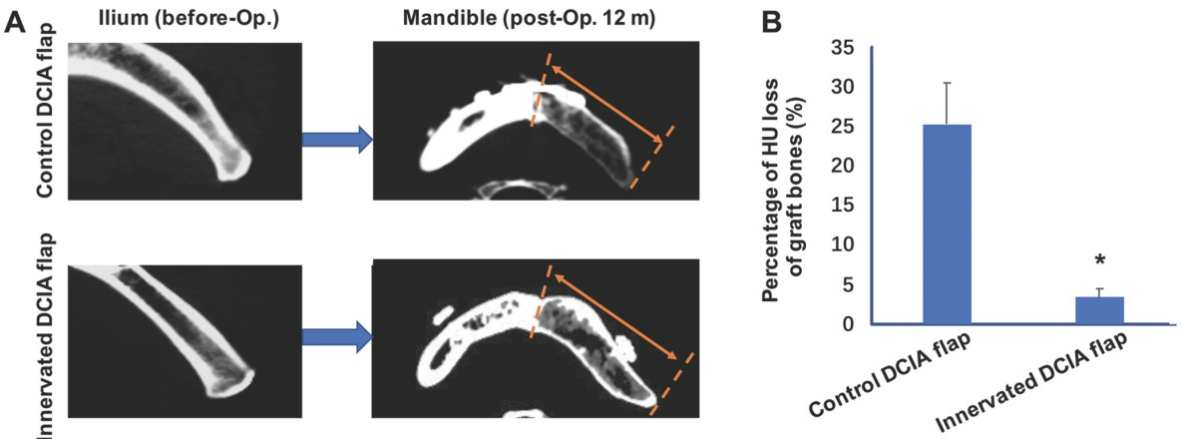
** Innervated deep circumflex iliac artery (DCIA) flap presented less bone resorption than control (non-innervated) DCIA flaps after mandibular reconstruction. (A)** Computed tomographic (CT) scans of the ilium right before grafted to segmental mandibular defects and the mandibles including the graft regions 12 months after bone reconstruction surgeries. The area between red broken lines and red arrows shows regions of iliac bone grafts. **(B)** Evaluations of graft bone resorption determined by calculating the percentage loss in graft bone in Hounsfield units (HU) of the CT scans. Significant decreases of bone density were found in innervated DCIA flaps when compared with non-innervated ones (*t* test; n=10 for each group, *p < 0.05).

In the reconstruction of the jaw, the commonly used vascularized iliac bone, the DCIA flap, often undergoes severe postoperative absorption, which affects the success of dental implants. In the preparation of a conventional iliac bone flap, the ilioinguinal nerve adjacent to the vascular pedicle is usually severed or sacrificed. The ilioinguinal nerve travelling *via* the internal oblique muscle is attached to the ilium and innervates the internal oblique muscle, but also innervates the ilium periosteum and bone marrow. Therefore, the reconstruction of sensory nerves may play an important role in maintaining the homeostasis of the bone graft. Our previous studies developed a method of using neurorrhaphy between the ilioinguinal nerve and the inferior alveolar nerve or auricular nerve during reconstruction of the mandibular bone 21. Moreover, this novel technique was applied in clinical setting with promising clinical results; it was found that 10/22 patients who underwent mandibular reconstruction with innervation, showed less bone resorption after 12 months at the CT scan and in histomorphometric analysis of the bone graft. Furthermore, it was identified that the bone quality around the dental implant was significantly higher compared with the non-innervated group. At 12 months after mandibular reconstruction, the Hounsfield unit (HU) loss of the grafted bone in the innervated group was significantly less compared with the non-innervated group (Figure 2) 21. Therefore, it was speculated that simultaneous innervation of a vascularized iliac bone graft can significantly reduce the risk of bone resorption after bone graft surgery. In addition, a clinical trial (Clinicaltrials.gov ID: NCT03889587) is currently underway, which includes a larger sample size of patients and there are other clinical trials and studies on bone regeneration or reduction of bone graft resorption (Table 2).

## Tissue engineered bone research on nerve regulating bone remodeling

As autologous bone grafts can cause damage and complications to surrounding bone and tissue in the donor site, there has been increasing research into tissue engineering of bone. Moreover, tissue-engineered bone is becoming a promising novel tool and contains four key elements: a scaffold with osteoconductivity; growth factors that induce osteogenesis; seeded cells with osteogenic potential; and tissue engineered vascularization or adequate blood supply 4. Previous studies have focused on advanced scaffold materials for bone regeneration 6, with cutting-edge research showing the importance of controlled release or molecularly imprinted intelligence for tissue regeneration or cancer treatment 1-5. In addition, previous studies have shown the importance of vascularization for tissue-engineered bone, especially early vascularization to provide nutrition for the formation of bone tissue 4, 96, 97. It has also been revealed that biomaterial alignment and optimized mechanical stimulation drive MSC differentiation, thus promoting osteogenesis *via* the stimulation of osteogenic cell recruitment to new bone formation areas 7, 98. Furthermore, studies have focused on the role of nerves in tissue engineered bone 53, 98, 99, and therefore, further research is required to investigate mature tissue-engineered products for the use in patients.

## Conclusions

**Figure 3:**
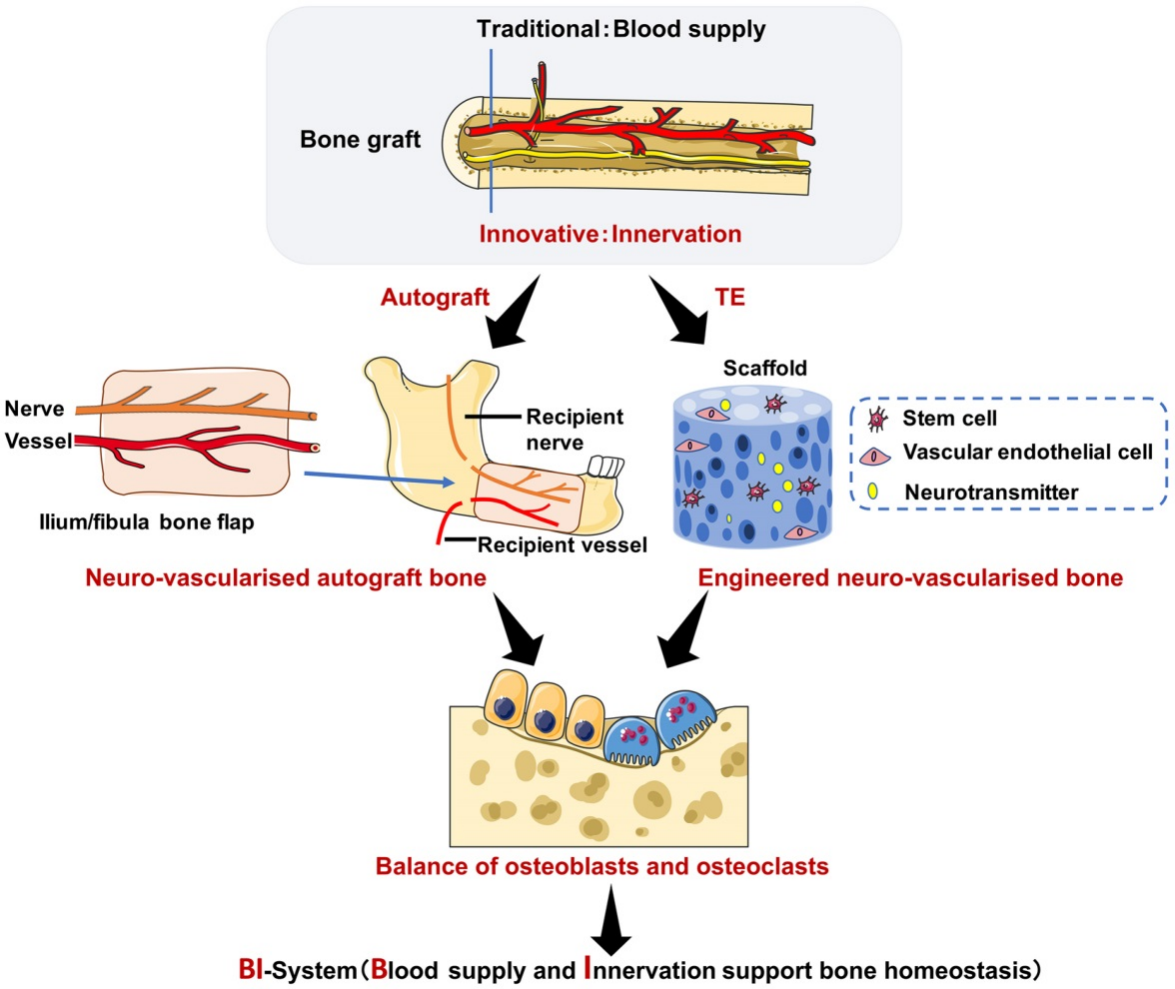
** A perspective in translational research of nerve-supported bone homeostasis.** On the one hand, a bony flap with both vascular anastomosis and neuroanastomosis to restore the mandibular defection leads to decreased post osteoporosis (the iliac/fibula bone graft is an example of autograft). On the other hand, stem cells derived from bone marrow, vascular endothelial cells and neurotransmitters all cultured in an artificial scaffold to reconstruct bone defection show balanced bone homeostasis. Abbreviations: TE: tissue engineering.

The role of central and peripheral nervous systems in bone remodeling has been proposed in the present review, and the latter system is of particular interest. Sensory and autonomic nerves regulate the peripheral nervous system *via* different types of neurotransmitters, but the specific mechanism is still elusive. Sensory nerves positively regulate bone remodeling *via* different sensory neurotransmitters acting on BMMSCs, such as CGRP, SP and Sema3A. Furthermore, sensory neurotransmitters can inhibit BMMSC aging and promote osteogenesis, which is of great significance for osteoporosis after clinical bone transplantation. Preliminary clinical studies have reported a positive role of innervation in maintaining the bone homeostasis in bone grafts. However, the innervation of bone graft requires further investigation. It is emphasized that there may be an important connection between the nervous system and bone remodeling, which will facilitate the development of bone grafting and tissue engineering. Furthermore, the novel application of nerve-based bone regeneration using BMMSCs provides a new insight in tissue engineering and clinical treatment of osteoporosis and other bone disorders (Figure 3).

## Figures and Tables

**Table 1:** Sensory and sympathetic neurotransmitters expressed in cells and tissues

Neurotransmitters	Expressed cells	Localization	Identification	References
NGF	Keratinocytes Mast cells Macrophages Osteoblasts Adipocyte	Bone (osteoblasts) Periosteal sensory nerves Adipose tissue	RT-PCR; Immunocytochemistry	54, 65, 66
CGRP	Sensory afferents Peptidergic primary Sensory neurons (spinal cord)	Bone/facture callus (BMSCs, Osteoblasts, Osteoclasts) Bone periosteum Bone trabecular Adipose tissue (Sensory nerve, Adipose stem cells) Endothelium (endothelial cells) Serum	RT-PCR; Immunocytochemistry; Immunostaining	14, 54, 56, 64, 71, 100
SP	Peptidergic sensory neurons Type B cells in the DRG Osteocytes	Articular cartilage/fracture callus/OA cartilage (Chondrocytes) Bone/facture callus (BMMSCs, BMSCs, BMMs) Wound region of bone (CD29+ MSCs) Osteoblasts Mature osteoclasts Immune cells Bone (bone periosteum, bone trabecular, epiphyseal growth plate, subchondral bone, ligaments, synovium) Adipose tissue (endothelial cells, immune cells) Blood vessel Smooth muscle Immune system (T- or B-lymphocytes, monocytes, mast cells, macrophages) Gland	In situ hybridization; Immunofluorescence; Immunoenzyme; RT-PCR; Immunohistochemistry	14, 18, 54, 56, 66, 76, 77, 101, 102
Sema3A	Axon Peripheral nerves Spinal cord Activated T cells Dendritic cells Angiogenic endothelial cells Bone cell lineages (chondrocytes, osteoblasts, and osteoclasts)	Bone (bone periosteum, bone marrow) Hypertrophic chondrocytes in ossification centers Endothelial cells Serum Brain Heart Lung Liver Fat	RT-PCR; Immunocytochemistry; Cytoenzymology; Immunostaining	84, 85, 86, 88, 103, 104
NE	Noradrenergic fibers Postsynaptic sympathetic neurons	Adipose tissue (efferent nerves, adipose stem cells, T cells, Macrophages) Osteoblasts	RT-PCR; Immunocytochemistry	54, 60

Abbreviations: DRG: dorsal root ganglion; NGF: nerve growth factor; CGRP, Calcitonin gene-related peptide; SP: substance P; Sema3A: Semaphorin 3A; NE: Norepinephrine; BMMSCs: bone marrow mesenchymal stem cells; BMSCs: bone marrow stromal cells; BMMs: bone marrow macrophages; RT-PCR: real-time polymerase chain reaction; OA: osteoarthritis.

**Table 2:** Clinical trials and researches on bone regeneration or reduction of bone graft resorption

Authors	Research	Intervention	Methods	Outcome
Gjerde et al	Clinical trial (NCT02751125)	Cell therapy (BMMSC) induced regeneration of severely atrophied mandibular bone	11 subjects (aged 52-79 years) with severe mandibular ridge resorption. Bone marrow cells were aspirated from the posterior iliac crest and plastic adherent cells were expanded in culture medium containing human platelet lysate. The MSCs and biphasic calcium phosphate granules as scaffolds were inserted subperiosteally onto the resorbed alveolar ridge.	The bone marrow cells were expanded *in vitro*. Significant new bone formation was induced. The regenerated bone volume was adequate for dental implant installation. Healing was uneventful, without adverse events.
Marrella et al 105	Biomaterial research	Engineering vascularized and innervated bone biomaterials for improved skeletal tissue regeneration	Highlight the structure and osteogenic functions of the vascular and nervous systems in bone, in a coupled manner. Discuss important design criteria for engineering vascularized, innervated, and neurovascularized bone implant materials.	Emphasised that bone implant materials with neurovascularized networks can more accurately mimic native skeletal tissue and improve the regeneration of bone tissue.
Wang et al 21	Clinical research	Preventing early-stage graft bone resorption by simultaneous innervation	Reported a new technique for simultaneous innervation of vascularized iliac flaps in mandibular reconstruction. 22 patients (aged 50 to 69 years) with postoncologic continuity defects of the mandible underwent mandibular reconstruction (10 innervated flaps and 12 control flaps).	Graft bone density loss in the control group was significantly higher than in the innervated group. Bone quality evaluation indicated a suitable condition for dental implantation in all patients in the innervated group. Histologic and histomorphometric analyses showed successful innervation in the innervated group but not in the control group. Osteoclast activity was significantly higher in the control group than in the innervated group.
Wang et al	Clinical trial (NCT03889587)	Innervation of vascularized iliac transplant avoids resorption in jaw bone reconstruction	Randomized controlled trial with 40 participants between the age of 17 to 65 years, irrespective of gender. Patients with post resection segmental defect of mandible between 5-9 cm long will be randomly assigned to 2 groups. Group 1 (Innervation)-There will be simultaneous innervation of vascularized iliac or fibular bone flaps through neurorrhaphy between the nerves innervating iliac or fibular bones and recipient site. Group 2 (Non-innervation)-This will be the traditional method of vascularized iliac or fibular bone flaps, and neurorrhaphy will not be performed.	The decreased ratio of the graft bone Hounsfield unit calculated by Spiral CT examination. It is used to reflect the degree of bone resorption. The index of successful innervated reconstruction. The innervation and sensation in the muscle island of innervated graft bone flap will be tested using neuroelectrophysiological and needling response examination. The graft bone samples taken by hollow drilling technique during the dental implant(s) procedure will be observed by silver staining.

Abbreviations: BMMSC: bone marrow mesenchymal stem cell; MSCs: mesenchymal stem cells.

## Abbreviations

BMMSC: Bone marrow mesenchymal stem cell
MSC: Mesenchymal stem cells
NGF: Nerve growth factor
SP: Substance P
CGRP: Calcitonin gene-related peptide
BMD: Bone mineral density
β-TCP: β-tricalcium phosphate
GVHD: Graft-versus-host disease
SLE: Systemic lupus erythematosus
HSCs: Hematopoietic stem cells
CD40L: CD40 ligand
FASL: Fas ligand
CTLA-4: Cytotoxic T‑lymphocyte protein 4
OPG: Osteoprotegerin
IFN-γ: Interferon γ
TNF-α: Tumor necrosis factor α
NFκB: Nuclear factor kappa B
PPARγ: Activated receptor gamma
Runx2: Runt-related transcription factor 2
M-CSF: Macrophage colony stimulating factor
SNS: Sympathetic nervous system
PSNS: Parasympathetic nervous system
NE: Norepinephrine
NPY: Neuropeptide Y
ACh: Acetylcholine
DO: Distraction osteogenesis
Col/nHA/Carr: Collagen/nano-hydroxyapatite/kappacarrageenan
TrkA: Tropomyosin receptor kinase A
Erk/Bcl-2: Extracellular signaling-regulated kinase/B-cell lymphoma-2
TRPV1: Transient receptor potential vanilloid 1
MAPK: Mitogen-activated protein kinase
NK1R: Neurokinin 1 receptor
BMMs: Bone marrow macrophages
Nrp: Neuropilin 1
Sema3A: Semaphorin 3A
PlxnA1: Plexin A1
TREM2: Triggering receptor expressed on myeloid cells 2
ITAM: Immune-receptor tyrosine-based activation motif
RhoA: Ras homolog family member A
Rac1: Rac family small GTPase 1
FARP2: FERM, ARH/RhoGEF and pleckstrin domain protein 2
β2-AR: Adrenoceptor beta 2
SDF-1: Stromal cell derived factor 1
MMP-2: Matrix metallopeptidase 2
TIMP-3: Tissue inhibitor of metalloproteinase 3
JNK: c-Jun N-terminal kinase
DCIA: Deep circumflex iliac artery
HU: Hounsfield unit
CT: Computed tomography
